# Cholangiocarcinoma in early childhood

**DOI:** 10.4322/acr.2024.504

**Published:** 2024-08-06

**Authors:** Amrit Kaur, Prakruthi S. Kaushik, Suma Mysore Narayana, Arun Kumar Ajjapanahalli Rajanna, Aruna Kumari Bandagadde Sreenivasa Bhat, Lingegowda Appaji

**Affiliations:** 1 Kidwai Memorial Institute of Oncology, Department of Pediatric Oncology, Bengaluru, Karnataka, India; 2 Kidwai Memorial Institute of Oncology, Department of Pathology, Bengaluru, Karnataka, India

**Keywords:** Pediatric Cancer, Cholangiocarcinoma, Jaundice, Ascites, Malignancy, Pediatric Oncology

To the Editor:

Cholangiocarcinoma (CCA) is a rare malignancy of the biliary tract, comprising 3% of all gastrointestinal cancers.^1^ Typically diagnosed in the seventh decade of life, it seldom occurs in the pediatric age group, making cases in pediatric patients notable.^2^ CCA in children and adolescents is frequently associated with underlying risk factors. In contrast, only 30% of adults exhibit them.^3^ We report the youngest presentation of a sporadic case of CCA in a 5-year-old boy with an unremarkable medical history.

A 5-year-old boy presented with a 15-day history of progressive abdominal distension and vomiting. Notably, there was no weight loss, fever, or jaundice. Clinical examination revealed a distended abdomen with dilated veins and marked hepatosplenomegaly. Laboratory findings included a hemoglobin of 12.5 g/dL (13-18 g/dl), a white blood cell count of 14,810/μL (4000-11500/uL), and a platelet count of 341,000/μL (331000/uL). The biochemical profile revealed a total serum bilirubin level of 0.6 mg/dL (0.2-1.2 mg/dL), serum aspartate aminotransferase of 37 U/L (up to 35 U/L), serum alanine transaminase of 11 U/L (up to 55 U/L), albumin of 3 g/dl (3.5-5.2 g/dL), and an INR of 1.1. Tests for human immunodeficiency virus, hepatitis B surface antigen, and hepatitis C virus were negative.

The abdominal and pelvic contrast-enhanced computed tomography revealed an enlarged liver with multiple scattered, well-defined hypodense lesions, the largest in segment VI of 3.3x3.0cm. The spleen was enlarged, without focal lesions. Multiple paraaortic and mesenteric lymph nodes were enlarged, and the abdomen had moderate free fluid. Serum carbohydrate antigen 19-9 (CA 19-9) was l136 U/mL (RR: 0-37 IU/mL), and alpha-fetoprotein was 2.19 IU/mL (RR: 0-8 IU/mL). An ultrasound-guided liver biopsy revealed a neoplasm characterized by tumor cells with eosinophilic cytoplasm, vesicular nuclear chromatin, and prominent nucleoli arranged in a glandular pattern, cords, and nests. Immunohistochemistry (IHC) results were strongly positive for CK7 and CK19 and negative for CK20, SALL4, AFP, Glypican 3, HepPar1, Arginase1, and β-catenin, suggesting CCA (Figure 1).

**Figure 1:**
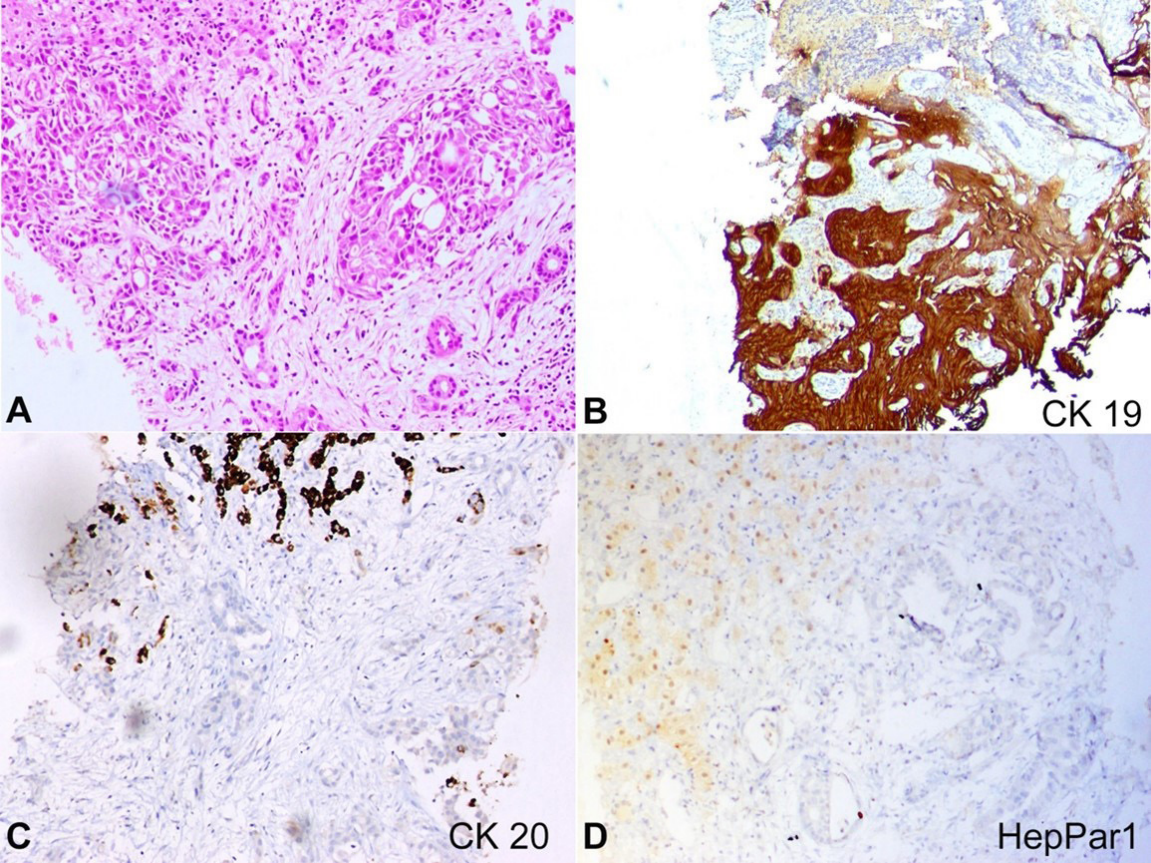
Photomicrographs of the liver biopsy. **A –** Microscopy displaying a neoplasm composed of tumor cells arranged in a glandular pattern, cords, and nests (H&E, 10X); **B –** positive, diffuse, and strong cytoplasmic reaction for CK19 (10X); **C –** negative reaction for CK 20 (10X); **D –** negative reaction for HepPar1(10X).

Fluorodeoxyglucose positron emission tomography (FDG-PET) indicated liver enlargement with FDG avid multiple hypodense lesions but no distant metastasis. Pediatric surgery consultation was sought, but due to the poor general condition of the patient and multifocal lesions in the liver, it was deemed inoperable. The child underwent systemic chemotherapy with gemcitabine and cisplatin. After two cycles, the patient developed massive pleural effusion, ascites, and respiratory failure. Repeat FDG PET indicated progressive disease, and the patient continued on supportive measures but ultimately succumbed to the illness. Parents were counselled to an autopsy to search for any underlying hepatic disease but were not willing to do the same.

CCA is an extremely rare malignancy in children, with an incidence of 0.0036/100,000, compared to that of 1.67/100,000 in the adult population.^4^ It is a bile duct malignancy and is divided into three subtypes depending on their anatomical site of origin: intrahepatic (iCCA), perihilar (pCCA), and distal CCA (dCCA). The most characteristic and common presentation of extrahepatic cholangiocarcinoma (eCCA) is jaundice and is seen only in 10-15% of cases of iCCA. iCCA is located in the liver parenchyma proximal to the second-degree bile ducts and generally presents with nonspecific symptoms like abdominal pain, nausea, weight loss, malaise, and night sweats. Diagnosis is often delayed in such cases. It also has a propensity for liver metastasis. Surgical resection with negative tumor margins is achieved only in 45% of the patients.^5^

Several risk factors have been linked to CCA like congenital biliary dilatation, choledochal cyst, choledocholithiasis, Caroli disease, primary sclerosing cholangitis (PSC), progressive familial intrahepatic cholestasis (PFIC), viral infections (Hepatitis B virus and hepatitis C virus), Inflammatory bowel disease, and *Opisthorchis viverrine* and *Clonorchis sinensis* infection.^6-8^ Most pediatric and adolescent cases have underlying risk factors for CCA, while only 30% have an underlying risk factor in adults. An extensive search revealed only three cases reported in English literature of pediatric cholangiocarcinoma presenting in the first decade of life. All cases had underlying risk factors; PFIC in 2 and 1 had congenital biliary dilatation. The presenting complaints were fever, pruritis, abdominal pain, and vomiting, and none of them had features of biliary obstruction. Two of them were diagnosed with iCCA and succumbed to their illness within 5 months of diagnosis. One had eCCA who underwent surgical resection and is alive post-year after surgery.^6,8^ To the best of our knowledge, our case represents the youngest presentation of sporadic CCA (Table 1).

**Table 1:** Summary of patients with pediatric cholangiocarcinoma presenting in the first decade of life described in the literature

Ref.	Age (years)/ Sex	Comorbidities	Presenting symptoms	Tumor markers	Site	Treatment	Status/ Follow up
Scheimann et al.^8^	4/F	PFIC	Fever	CA 19-9 132 IU/mL	ICCA	Chemo	Death within 5 months from diagnosis
		Giant cell hepatitis					
		Biliary cirrhosis		AFP-61.5 IU/mL			
Scheimann et al.^8^	7/F	PFIC	Pruritis	Not available	ICCA	None	Death within 4 months from diagnosis
		Giant cell hepatitis					
		Biliary cirrhosis					
Saikusa et al.^6^	3/M	Congenital biliary dilatation with pancreaticobiliary maljunction	Abdominal pain	Not available	ECCA	Resection	Alive/ 1 year
Index case	5/M	No comorbidities	Abdominal distension	CA 19-9- 136 IU/mL	ICCA	Chemo	Death within 2 months from diagnosis
				AFP (–) 2.19IU/mL			

AFP: Alpha-fetoprotein. CA: Carbohydrate antigen; Chemo: chemotherapy; ECCA: Extrahepatic cholangiocarcinoma; ICCA: Intrahepatic cholangiocarcinoma; PFIC: Progressive familial intrahepatic cholestasis.

Morphologically, CCA can be tubular/acinar adenocarcinoma with well, moderate, or poor differentiation and show immunopositivity for CK7, CK19, and EMA. Metastatic colorectal adenocarcinoma, upper gastrointestinal tract cancers, and metastatic pancreaticobiliary adenocarcinoma are close differentials and can be differentiated based on CK20 and a comprehensive IHC panel including MUC2, MUC5AC, CA19-9, mCEA, CA125, SMAD4, respectively.^9^

The prognosis for children and adolescents with CCA is unfavorable. Surgery is a potentially curative option; however, most patients have metastatic or locally advanced disease at presentation, and only 25% are eligible for resection. Robust data supports the use of first-line cisplatin and gemcitabine chemotherapy in adults with advanced disease.^10^ Survival is related to the extent of disease spread; thus, early diagnosis of CCA is essential for positive outcomes.

## References

[1] Khan SA, Thomas HC, Davidson BR, Taylor-Robinson SD (2005). Cholangiocarcinoma. Lancet.

[2] Jesper D, Heyn SG, Schellhaas B (2018). Effects of liver cirrhosis and patient condition on clinical outcomes in intrahepatic cholangiocarcinoma: a retrospective analysis of 156 cases in a single centre. Eur J Gastroenterol Hepatol.

[3] Banales JM, Marin JJG, Lamarca A (2020). Cholangiocarcinoma 2020: the next horizon in mechanisms and management. Nat Rev Gastroenterol Hepatol.

[4] Newsome JR, Venkatramani R, Heczey A, Danysh HE, Fishman DS, Miloh T (2018). Cholangiocarcinoma among children and adolescents: a review of the literature and surveillance, epidemiology, and end results program database analysis. J Pediatr Gastroenterol Nutr.

[5] Valle JW, Borbath I, Khan SA, Huguet F, Gruenberger T, Arnold D (2016). Biliary cancer: ESMO Clinical Practice Guidelines for diagnosis, treatment and follow-up. Ann Oncol.

[6] Saikusa N, Naito S, Iinuma Y, Ohtani T, Yokoyama N, Nitta K (2009). Invasive cholangiocarcinoma identified in congenital biliary dilatation in a 3-year-old boy. J Pediatr Surg.

[7] Tyson GL, El-Serag HB (2011). Risk factors for cholangiocarcinoma. Hepatology.

[8] Scheimann AO, Strautnieks SS, Knisely AS, Byrne JA, Thompson RJ, Finegold MJ (2007). Mutations in bile salt export pump (ABCB11) in two children with progressive familial intrahepatic cholestasis and cholangiocarcinoma. J Pediatr.

[9] Vij M, Puri Y, Rammohan AGG (2022). Pathological, molecular, and clinical characteristics of cholangiocarcinoma: a comprehensive review. World J Gastrointest Oncol.

[10] Valle J, Wasan H, Palmer DH (2010). Cisplatin plus gemcitabine versus gemcitabine for biliary tract cancer. N Engl J Med.

